# Supplementary material for: Hotspots of dendritic spine turnover facilitate clustered spine addition and learning and memory
Source: Nat Commun. 2018 Jan 29;9:422. doi: 10.1038/s41467-017-02751-2 (PMC5789055; doi:10.1038/s41467-017-02751-2)
Supplement: Supplementary file 1 — Supplementary Information [file 41467_2017_2751_MOESM1_ESM.pdf]

Supplementary Figure 1

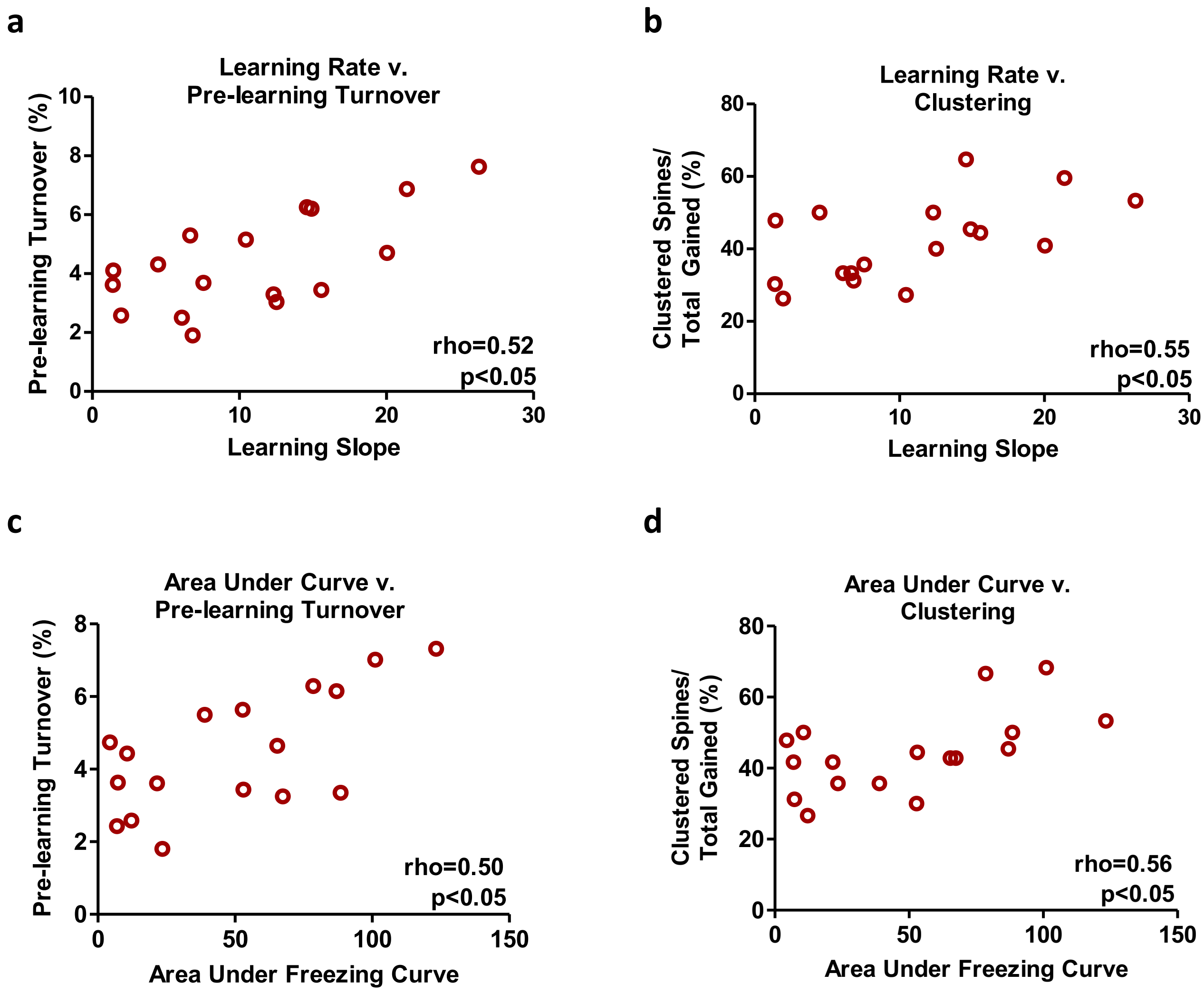


**Supplementary Figure 1. Pre-learning spine turnover and learning-related clustering correlate with the rate of learning.** (**a**) Dendritic spine turnover rate before training correlates with future rate of contextual learning. Scatter plot showing the relationship between dendritic spine turnover prior to training (Day -3 to Day 0) and the rate of learning during the contextual conditioning task ($p$=0.0307). (**b**) Clustered spine formation is linearly correlated with the rate of learning. Scatter plot showing the relationship between dendritic spine clustering and the rate of learning during the contextual conditioning task ($p$=0.0212). (**c**) Dendritic spine turnover rate before training correlates with future contextual learning. Scatter plot shows the relationship between dendritic spine turnover prior to training (Day-3 to Day0) and the area under the curve of each mouse's contextual freezing curve ($p$=0.0401). (**d**) Clustered spine formation is linearly correlated with contextual fear learning. Scatter plot shows the relationship between dendritic spine clustering and the area under the curve of each mouse's contextual freezing curve ($p$=0.0197). Each circle represents a single mouse ($n$=17 mice). Spearman's rho is indicated in **a** through **d**.

Supplementary Figure 2

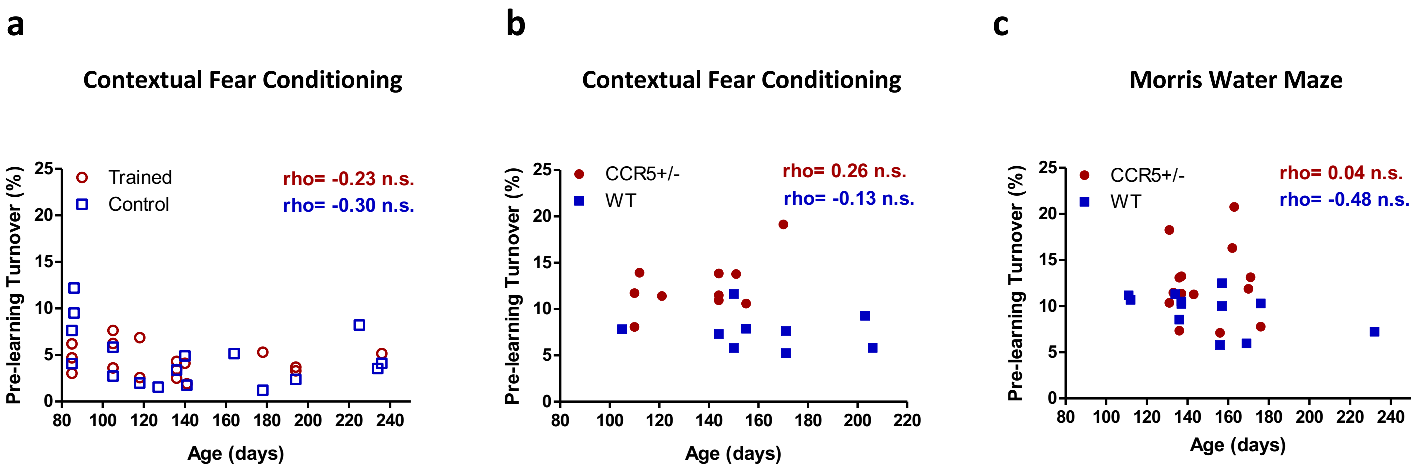


**Supplementary Figure 2. Pre-training spine turnover is not affected by age between 3 to 8 months.** (**a**) Baseline spine turnover rates in CFC for WT trained and home-caged mice are not correlated with the age at the first day of imaging (Trained *n*=17, *p*=0.3747; control *n*=17, *p*=0.2407). (**b, c**) Baseline spine turnover rate is not correlated with the age at the first day of imaging in contextual (**b**, *Ccr5*$^{+/-}$ *n*=10, *p*=0.4697; WT *n*=9, *p*=0.7435) or spatial (**c**, *Ccr5*$^{+/-}$ *n*=14, *p*=0.8810; WT *n*=12, *p*=0.1137) learning groups. Spearman's rho is indicated in **a**, **b** and **c**. n.s., not significant.

Supplementary Figure 3

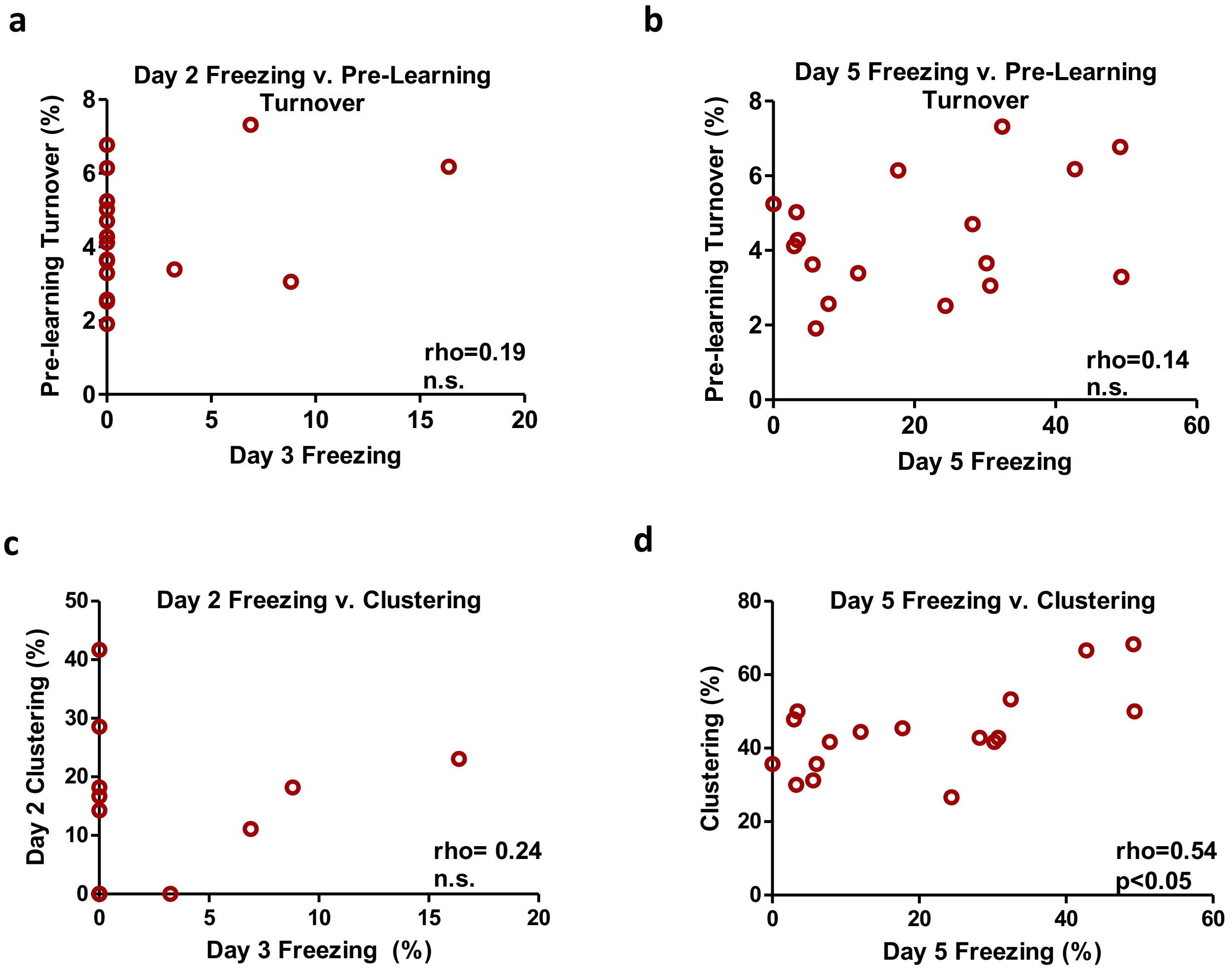


**Supplementary Figure 3. Analysis of the relationship of freezing to clustering and pre-learning turnover during contextual fear conditioning.** (**a**) Dendritic spine turnover rate before training does not correlate with contextual learning on day 2 of training. Scatter plot shows the relationship between dendritic spine turnover prior to training and contextual freezing on day 2 ($p$=0.4627). (**b**) Dendritic spine turnover rate before training does not correlate with contextual learning on day 5 of training. Scatter plot shows the relationship between dendritic spine turnover prior to training and contextual freezing on day 5 ($p$=0.5798). (**c**) Clustered spine formation by day 2 of training is not correlated with freezing. Scatter plot shows the relationship between dendritic spine clustering and contextual freezing on day 2 ($p$=0.3530). (**d**) Clustered spine formation is linearly correlated with contextual freezing on day 5 of training. Scatter plot shows the relationship between dendritic spine clustering and contextual freezing on day 5 ($p$=0.0239). Each circle represents a single mouse ($n$=17 mice). Spearman's rho is indicated in **a** through **d**. n.s., not significant.

Supplementary Figure 4

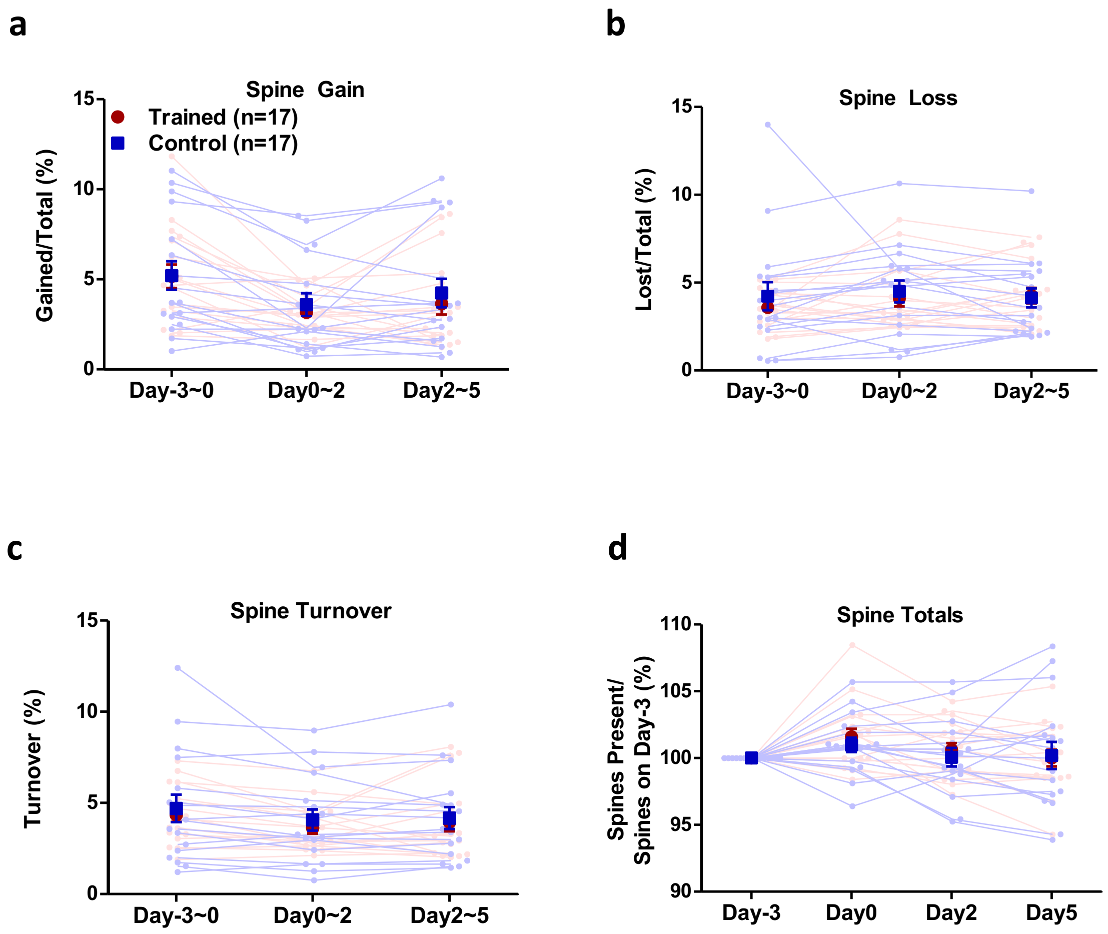


**Supplementary Figure 4. Spine gain and loss are not altered by contextual learning**. (**a**) The number of spines formed across imaging sessions does not differ between trained and control mice. Each point represents the number of spines formed between two time points divided by the total number of spines present at the first time point (i.e. Day -3~0 is the number of new spines present on Day 0 divided by the total number of spines present on Day -3; Trained vs Control, Day -3~0 = 5.2% vs 5.2%; Day 0~2= 3.2% vs 3.6%; Day 2~5= 3.6% vs 4.2%). (**b**) The number of spines lost across imaging sessions does not differ between groups. Each point is the number of spines lost between two time points divided by the total number of spines present at the first time point (i.e. Day -3~0 is the number of spines lost by Day 0 divided by the total number of spines present on Day -3; Trained vs Control, Day -3~0= 3.6% vs 4.2%; Day 0~2= 4.1% vs 4.5%; Day 2~5= 4.3% vs 4.1%). (**c**) The spine turnover observed is not affected by learning. Percentage of turnover is the sum of the number of newly formed and lost spines between two time points divided by the sum of the total number of spines across both time points (Trained vs Control, Day -3~0 = 4.3% vs 4.7%; Day 0~2= 3.6% vs 4.1%; Day 2~5= 3.9% vs 4.2%). (**d**) The total number of spines observed does not differ between groups across the course of the experiment (Trained vs Control, Day -3=100% vs 100%; Day 0=101.6% vs 101.0%; Day 2=100.6% vs 100.1%; Day 5=100.0% vs 100.2%). Data are represented as mean ± s.e.m. Trained $n$=17, control $n$=17 mice; $p$>0.05 for **a**, **b**, **c** and **d**. Mann Whitney *U-test*; two-tailed $p$-value corrected for multiple comparisons.

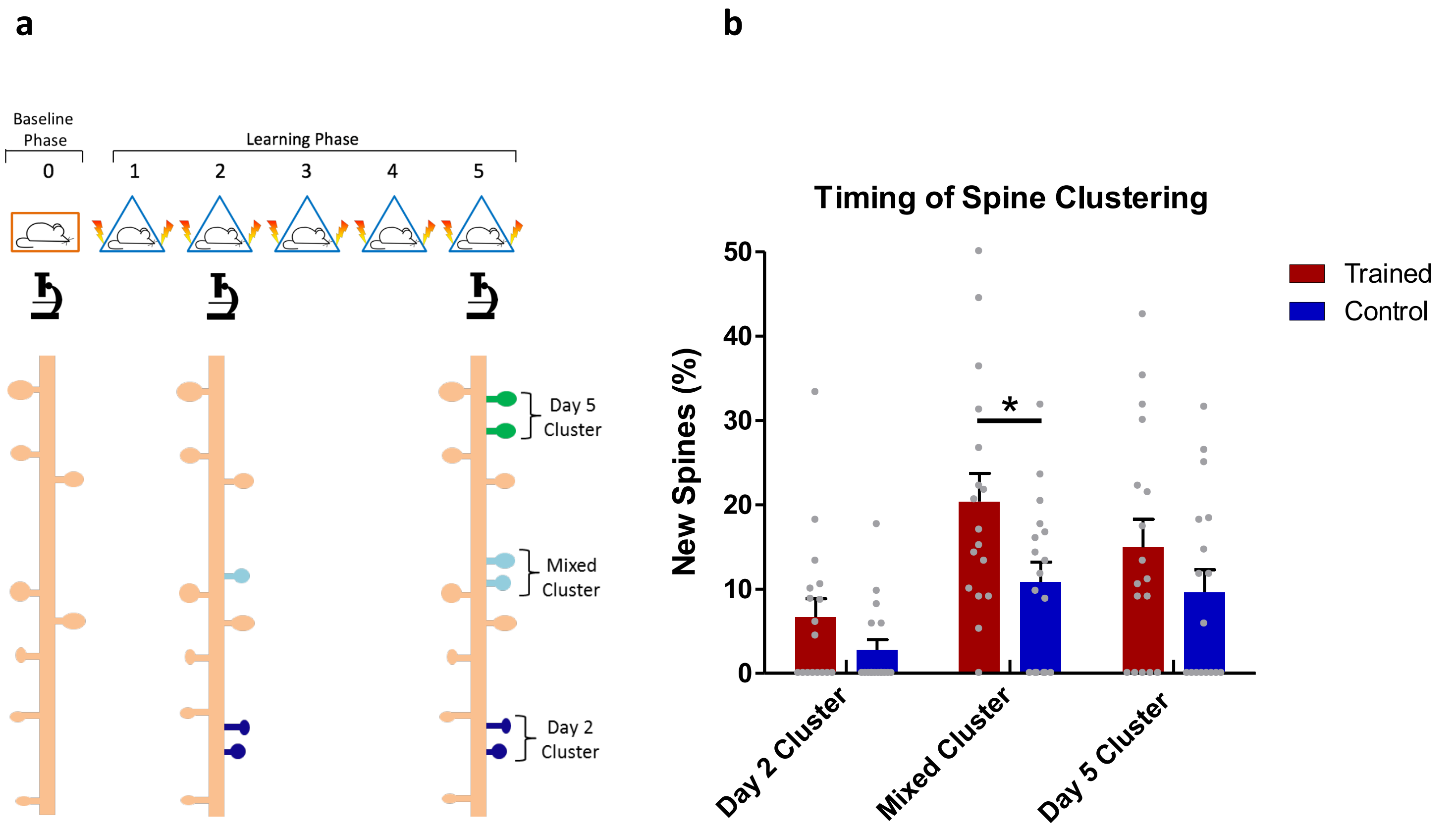


**Supplementary Figure 5. Spine clustering increases with subsequent training during contextual learning.** (**a**) Schematic representation of clustered spine addition throughout training. Day 2 clusters are created when two or more new spines are added within 5 µm of each other by the second day of training and persist to the end of training (dark blue spines). Mixed clusters are created when one spine is added by the second day of training and other new spines are added within 5 µm of each other by the final day of training (light blue spines). Day 5 clusters are created when two or more new spines are added within 5µm of each other by the final day of training (green spines). (**b**) Timing of clustering and distribution of cluster types for trained and control mice. For trained mice 42.0% of new spines formed clusters: 6.7% formed a cluster by Day 2; 20.4% formed in mixed clusters; 14.9% formed a cluster by day 5. For control mice 23.0% of new spines formed clusters: 2.8% occurred by day 2, 10.8% occurred in mixed clusters, and 9.6% occurred in day 5 clusters. Trained mice have more learning-related spines that form in clusters, and have significantly more new spines formed as mixed clusters ($n$=17 mice per group; Two-way RM ANOVA, training x time interaction $F_{(2,64)}$=0.51, $p$=0.6034; Training effects $F_{(1,32)}$=16.98, $p$=0.0002; Time effects $F_{(2,64)}$=7.27, $p$=0.0014; Bonferroni post-tests for trained and control in mixed cluster: $p$<0.05). New spines are those formed during the learning phase and persistent until training ends. *$p$<0.05.

Supplementary Figure 6

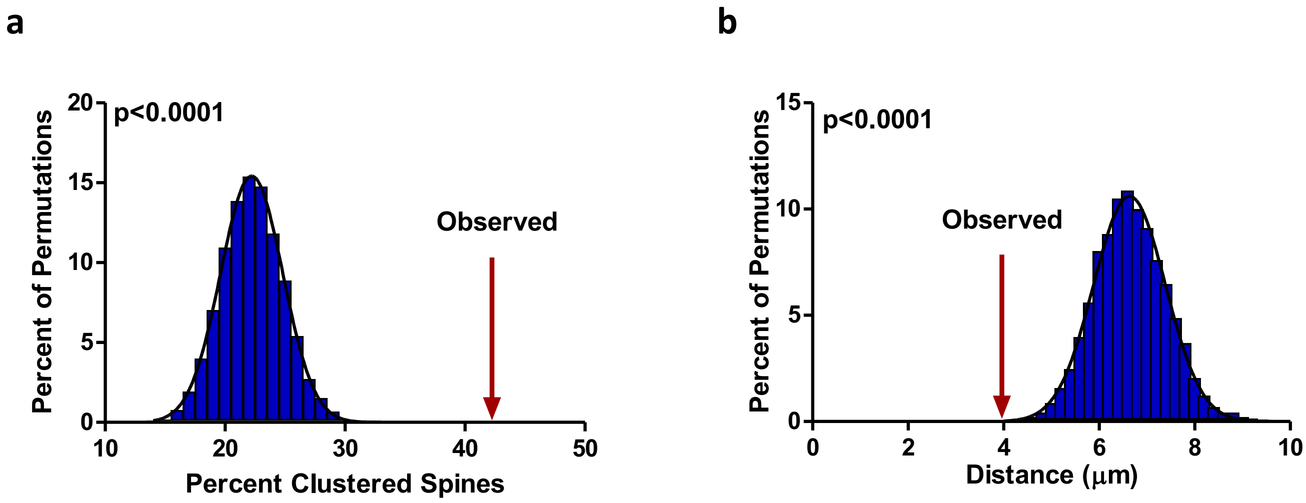


**Supplementary Figure 6. Permutation analysis of percent spine clustering and average clustering-to-turnover distance yields similar results as randomized simulations.** (**a**) The percentage of clustered new spines is significantly greater than chance. Shown is a histogram of 10,000 permutations of new spine identity, where the percent of new spines within 5 μm of each other was calculated. The arrow represents the actual percentage of clustered spines observed from the data (42%). Black line is Gaussian fit of data (mean of Gaussian fit = 22.2%, observed= 42.0%, *n*=17 mice; *p*<0.0001, as no permutation values were as or more extreme than the observed value). (**b**) The average nearest neighbor distance from each learning-related clustered spine to its closest neighboring pre-learning turnover spine is significantly smaller than chance. 10,000 permutations of pre-learning turnover spine identity were run, with nearest neighbor distance from clustered spines to turnover spines measured and averaged in each. The arrow represents the actual average nearest neighbor distance (4.0 μm) from each learning-related clustered spine to its closest neighboring pre-learning turnover spine observed in the data. Black line is Gaussian fit of data (mean of Gaussian fit = 6.6 μm, *n*=152 distance measurements; *p*<0.0001, as no permutation values were as, or more extreme than our observed value).

Supplementary Figure 7

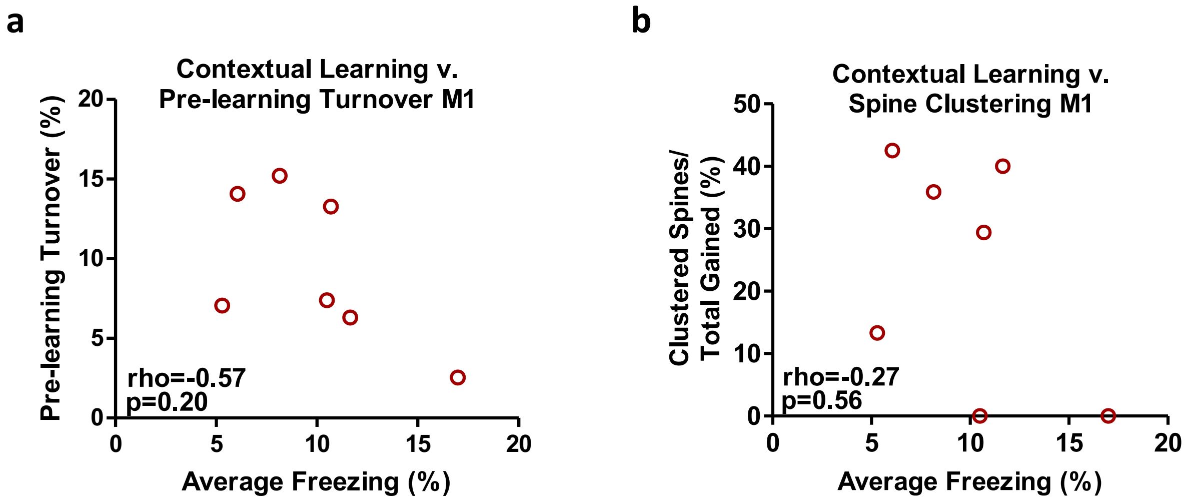


**Supplementary Figure 7. Spine dynamics in primary motor cortex are not influenced by contextual learning.** (**a**) Baseline spine turnover in M1 prior to training is not correlated with future contextual learning ($p$=0.20). (**b**) The percentage of new spines added in clusters in primary motor cortex does not correlate with the level of contextual learning ($p$=0.56). Spearman's rho is indicated in **a** and **b**.

Supplementary Figure 8

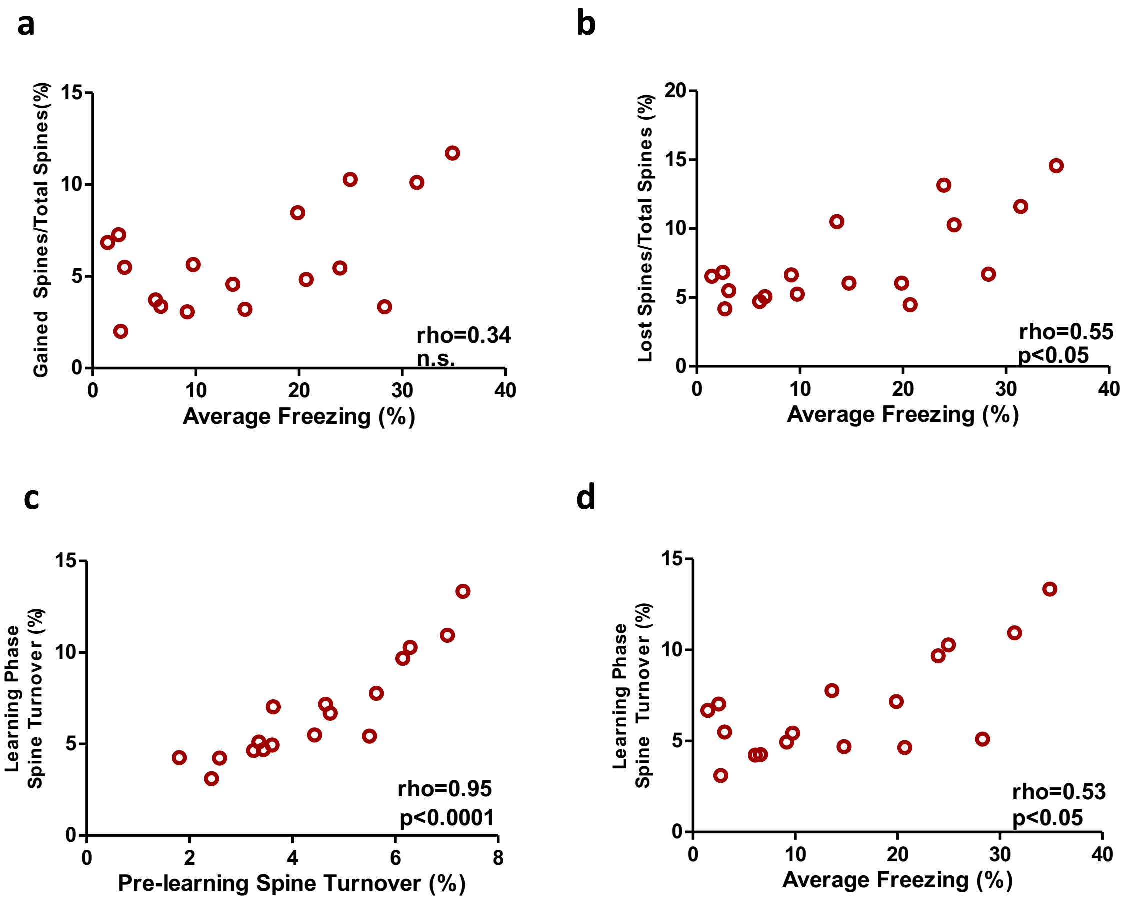


**Supplementary Figure 8. Learning is not correlated to spine gain, but does correlate with spine loss and spine turnover during learning.** (**a**) Total spine gain (as a percentage of the total number of spines) does not correlate with average freezing ($n$=17 mice; *Spearman's rho*=0.34, $p$=0.18). (**b**) Spine loss during contextual learning correlates with average freezing ($n$=17 mice; *Spearman's rho*=0.55, $p$=0.023). (**c**) Pre-training spine turnover is significantly correlated with learning phase spine turnover ($n$=17 mice; *Spearman's rho*=0.95, $p$<0.0001). (**d**) Learning phase spine turnover correlates with average freezing ($n$=17 mice; *Spearman's rho*=0.53, $p$=0.028). n.s., not significant.

Supplementary Figure 9

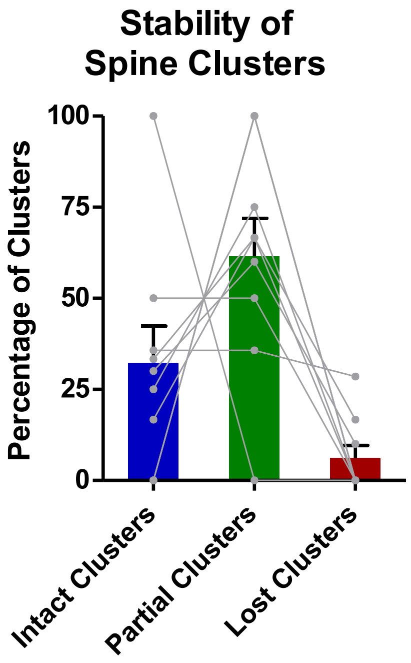


**Supplementary Figure 9. Spine clusters are stable after training.** Analysis of the stability of clusters of spines at 4-6 weeks following training reveals that 32.3% of clusters are fully intact without any loss of spines. Only 6.1% of clusters are completely lost, with all spines initially forming the cluster subsequently lost by 4-6 weeks. Finally, 61.6% of clusters have one or more spines stable by 4-6 weeks, but not all spines initially forming the cluster are present at this remote imaging time point (*n*=9 mice).

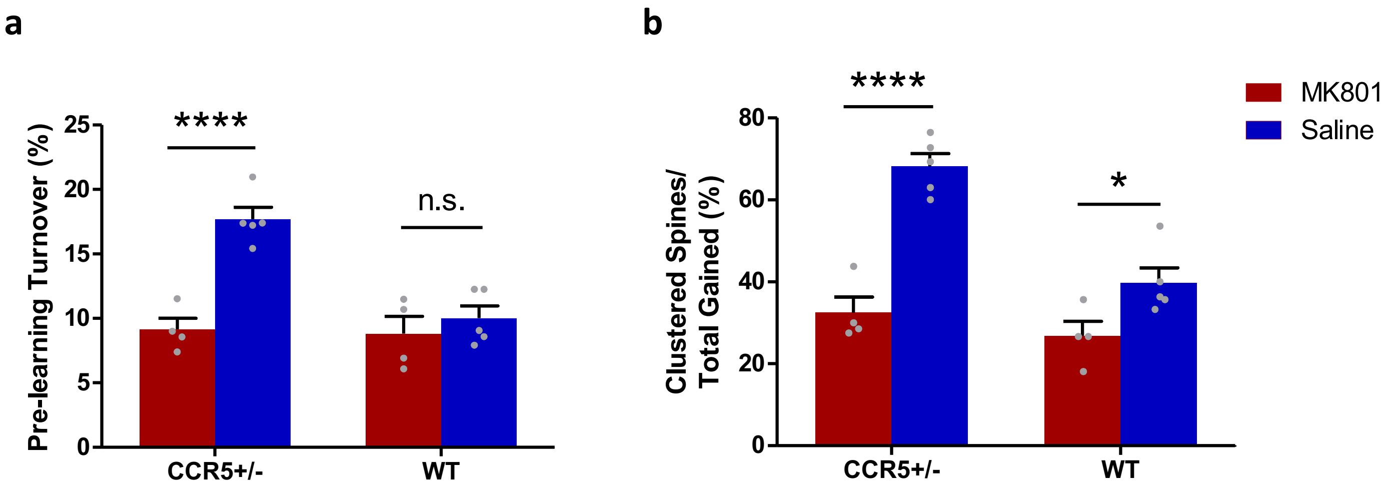


**Supplementary Figure 10. Enhanced pre-learning spine turnover and learning-related spine clustering in *Ccr5*$^{+/-}$ mice depend on NMDA receptor activity.** (**a**) *Ccr5*$^{+/-}$ mice that receive MK801 treatment (0.25 mg/kg, twice daily) no longer have enhanced baseline spine turnover rate, while MK801 does not have a significant effect on their WT littermates (MK801-*Ccr5*$^{+/-}$ *n*=4, Saline-*Ccr5*$^{+/-}$ *n*=5, MK801-WT *n*=4, Saline-WT *n*=5, Two-way ANOVA, genotype x treatment interaction: $F_{(1,14)}$=13.00, *p*=0.0029; Bonferroni post-test for *Ccr5*$^{+/-}$ : *p*<0.0001, WT: *p*>0.05). (**b**) MK801 treatment significantly reduce contextual fear conditioning-related clustered spine formation in both *Ccr5*$^{+/-}$ and WT littermates (MK801-*Ccr5*$^{+/-}$ *n*=4, Saline-*Ccr5*$^{+/-}$ *n*=5, MK801-WT *n*=4, Saline-WT *n*=5, Two-way ANOVA, genotype x treatment interaction: $F_{(1,14)}$=10.51, *p*=0.0059; Bonferroni post-test for *Ccr5*$^{+/-}$ : *p*<0.0001, WT: *p*<0.05). Data are represented as mean ± s.e.m. ****p<0.0001, *p<0.05, n.s., not significant.

Supplementary Figure 11

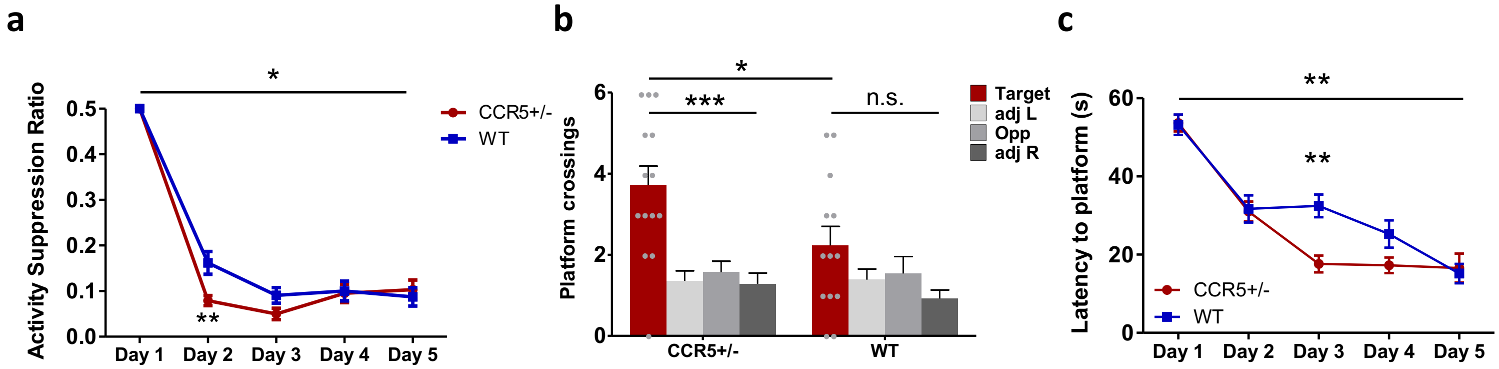


**Supplementary Figure 11**. ***Ccr5*$^{+/-}$ mice show enhanced learning and memory in contextual and spatial learning tasks**. (**a**) *Ccr5*$^{+/-}$ mice have enhanced activity suppression after one day of training in CFC. Activity suppression ratio is the average activity during testing divided by the sum of baseline activity plus activity during testing (*Ccr5*$^{+/-}$ $n$=12, WT $n$=15; Two-way RM ANOVA, genotype x time interaction: $F_{(4,100)}$=2.74, $p$=0.0329; Bonferroni post-test for Day2: $p$<0.01). (**b**) In a MWM probe test given after 3 days of training, *Ccr5*$^{+/-}$ mice show enhanced accuracy for recall of platform location compared to the WT littermates, as shown by the increased number of crossings of the platform location (*Ccr5*$^{+/-}$ $n$=14, WT $n$=13; Two-way RM ANOVA, genotype x platform crossings in each quadrant interaction $F_{(3,75)}$=2.04, $p$=0.1153; Bonferroni post-tests for the target quadrant versus all other quadrants: $p$<0.001 for *Ccr5*$^{+/-}$, $p$>0.05 for WT; Unpaired $t$-test for the target quadrant, $t_{(25)}$=2.221, $p$=0.0356). (**c**) *Ccr5*$^{+/-}$ mice show enhanced learning rate in MWM task compared to the WT littermates, as shown by the faster decrease of latency to platform (latency indicates the average time spent in searching for the platform across 4 trials per training day; *Ccr5*$^{+/-}$ $n$=14, WT $n$=13; Two-way RM ANOVA, genotype x time interaction: $F_{(4,100)}$=3.89, $p$=0.0056; Bonferroni post-test for Day3: $p$<0.01). Data are represented as mean ± s.e.m. ***$p$<0.001, **$p$<0.01, *$p$<0.05; n.s., not significant.

Supplementary Figure 12

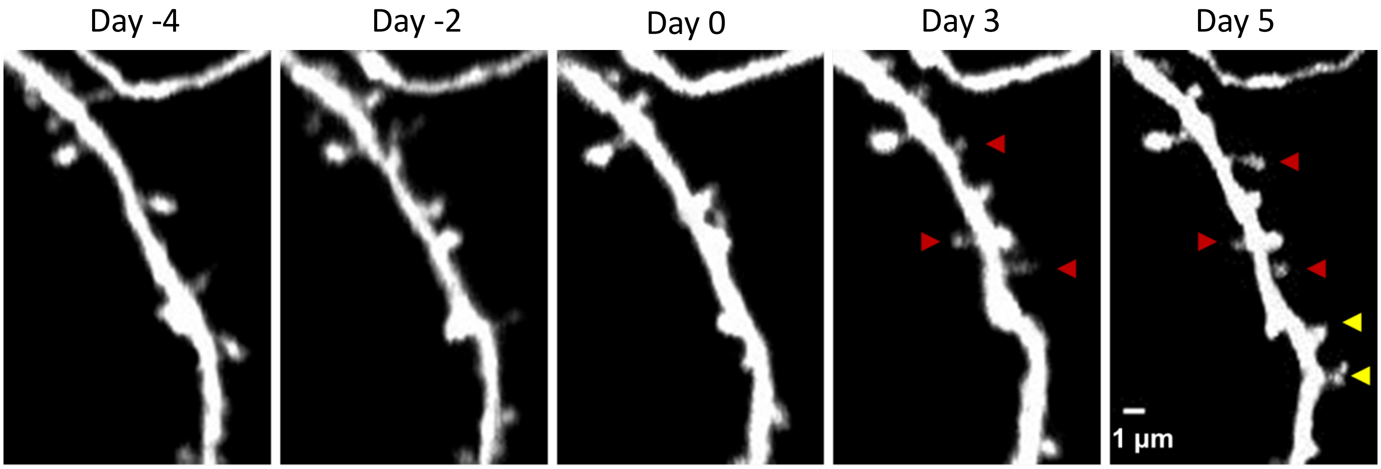


**Supplementary Figure 12. Representative example of longitudinal imaging of a dendritic segment during spatial learning.** Three new spines were added by Day 3 of MWM training within 5 μm of each other (red arrowhead). Two new spines were added between Day 3 and 5 of training within 5 μm of each other (yellow arrowhead). There are total five learning-related clustered spines in this example. Scale bar indicates 1 μm.

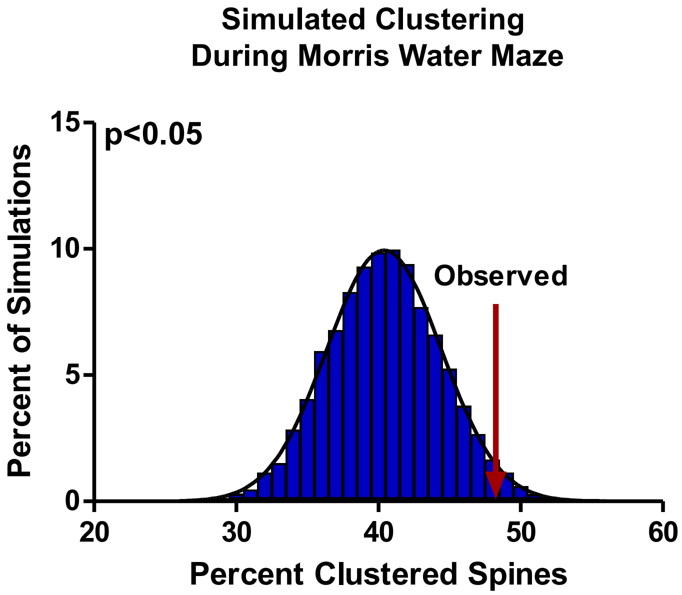


**Supplementary Figure 13. Resampling analysis indicates that the percentage of clustered new spines during spatial learning is significantly greater than chance.** Shown is a histogram of 10,000 simulations of randomized new spine positions, where the percent of new spines within 5 µm of each other was calculated and averaged across mice. The arrow represents the actual averaged percentage of clustered spines observed from the MWM data. Black line is Gaussian fit of data (mean of Gaussian fit = 40.4%, observed= 48.8%, $n$=7 mice; $p$=0.0185, one side).

Supplementary Figure 14

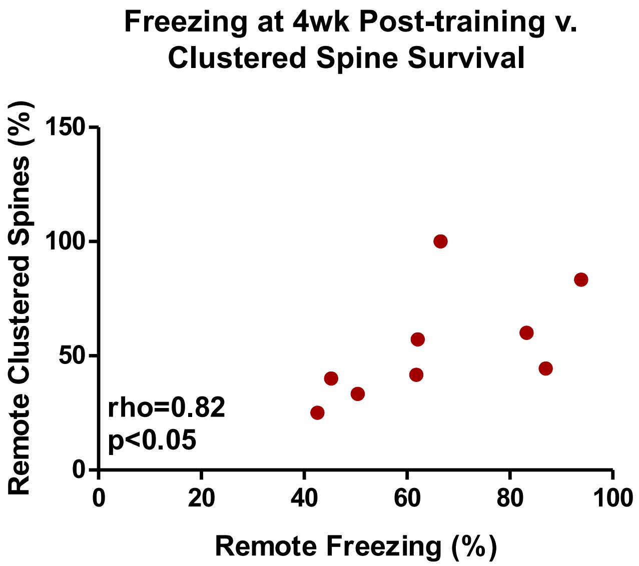


**Supplementary Figure 14. The percentage of persistent learning-related clustered spines is correlated with remote memory.** Wild type mice were tested for their memory of the training context four weeks after contextual training ended. mice were re-imaged at this time point and the stability of clustered spines formed during learning was assessed. The number of surviving clustered spines was divided by the total number of surviving spines formed during learning to calculate the percentage of remote clustered spines. This percentage is linearly correlated with the freezing levels at 4 weeks post-training ($n$=9 WT mice; $p$=0.0108). Spearman's rho is indicated on graph.

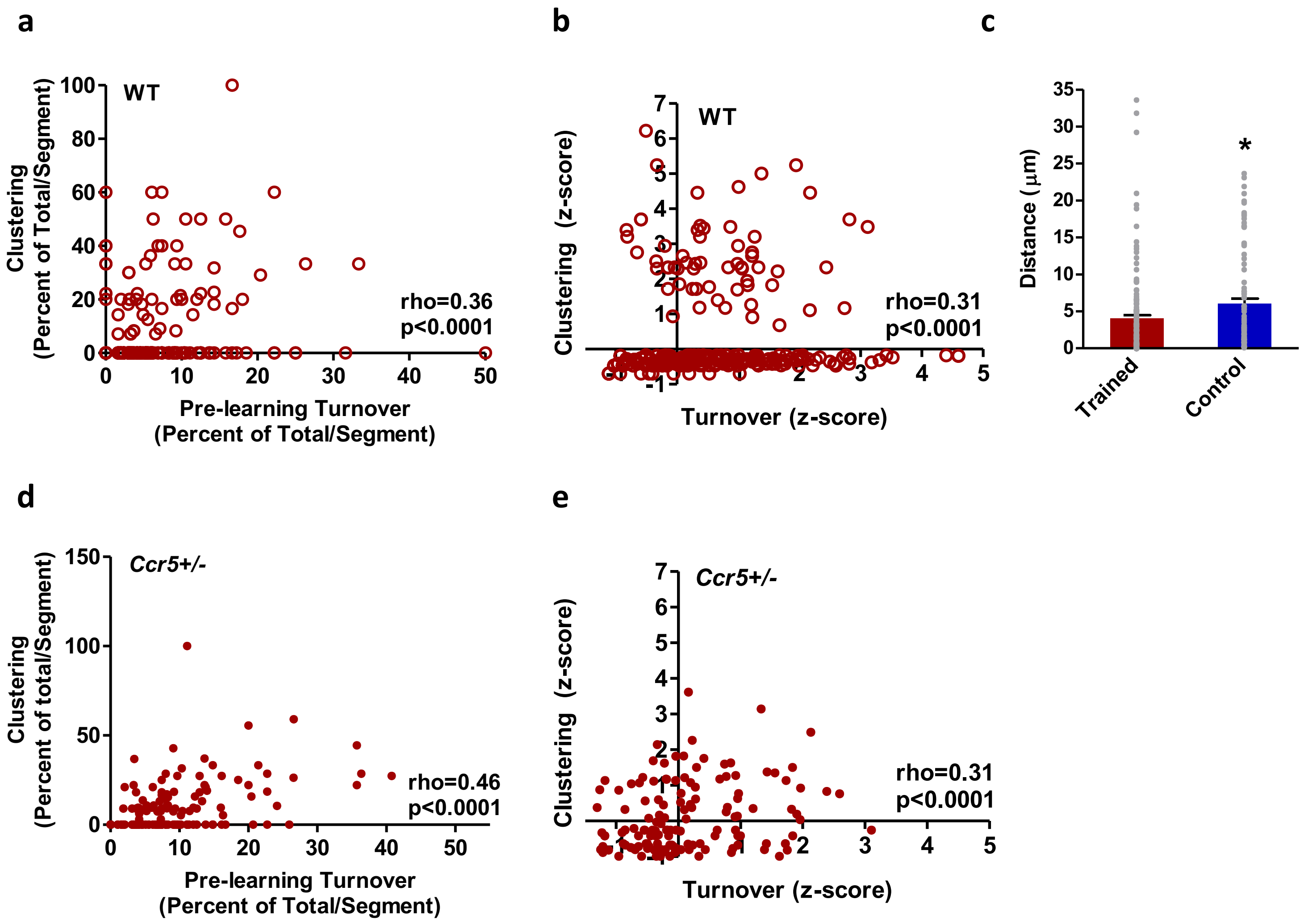


**Supplementary Figure 15. Pre-learning turnover and learning-related spine clustering by individual dendritic segments are correlated.** (**a**) Pre-learning spine turnover and clustering by dendritic segment are significantly correlated in trained WT mice. For each dendritic segment, turnover and clustering were calculated as a percentage of the total for a given mouse ($n$=577 segments over 17 mice, $p$<0.0001). (**b**) Standardized pre-learning turnover and learning-related clustering by dendritic segment are significantly correlated. Turnover and clustering for each segment were standardized across mice via z-scoring ($n$=577 segments over 17 mice, $p$<0.0001). (**c**) The average distance between clustered spines and the nearest turnover spine is significantly smaller in trained versus control mice. (Trained $n$=152 distance measurements, average distance=4.0 μm; n=91 control measurements, average distance=6.0 μm; *Mann-Whitney U*=5709, $p$=0.0188). (**d**) Pre-learning spine turnover and clustering by dendritic segment are significantly correlated in trained *Ccr5*$^{+/-}$ mice. For each dendritic segment, turnover and clustering were calculated as a percentage of the total for a given mouse ($n$=157 segments over 14 mice, $p$=0.0117). (**e**) Standardized pre-learning turnover and learning-related clustering by dendritic segment are significantly correlated in trained *Ccr5*$^{+/-}$ mice. Turnover and clustering for each segment were standardized across mice via z-scoring ($n$=157 segments over 14 mice, $p$<0.0001). Spearman's rho is indicated in **a, b, d** and **e**.

Supplementary Figure 16

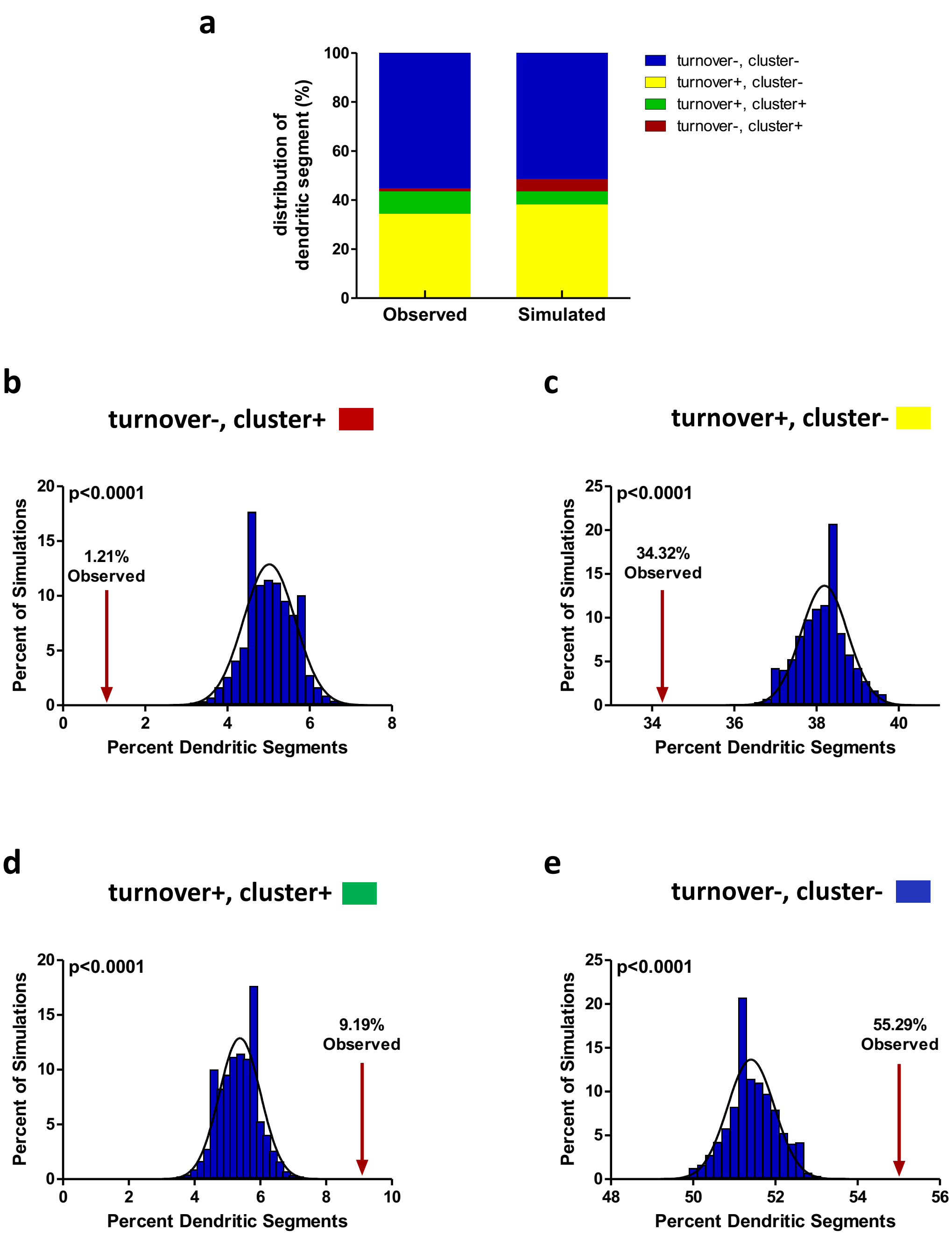


**Supplementary Figure 16. Resampling analysis indicates that the distribution of dendritic segments with levels of pre-learning turnover and post-learning clustering observed in the data is greater than chance.** (**a**) Observed distribution of dendritic segments that fall into the four categories: i. segments with no pre-learning turnover spines or clustered spines (blue); ii. segments with pre-learning turnover, but without clustered spines after learning (yellow); iii. segments with both pre-learning turnover and clustered spines (green); iv. segments without pre-learning turnover spines, but gain clustered spines after learning (red). Each observed percentage of the 4 categories is shown individually in the following graphs. The distribution of segments in the 4 categories are calculated using simulated values, which is done by permuting the number of clustered spines on each dendritic segment within each subject and recalculating the percentages of segments of the 4 categories. Simulated distribution is the average of simulated values of 17 trained mice. (**b, c**) Percentage of segments without turnover, but gain clusters (**b**) and segments with turnover but do not gain clusters (**c**) are both lower than chance level. (**d, e**) Percentage of segments with both turnover and clusters (**d**) and segments without any turnover or clusters (**e**) are both higher than chance level. Shown in **b,c,d** and **e** is a histogram of 10,000 permutations. The arrow represents the actual percentage of dendritic segments in indicated category observed from the data. Black line is Gaussian fit of data (mean of Gaussian fit = 5.0%, 38.2%, 5.4% and 51.4% in **b,c,d** and **e** respectively; observed values are indicated in graphs; *n*=17 trained mice).

Supplementary Figure 17

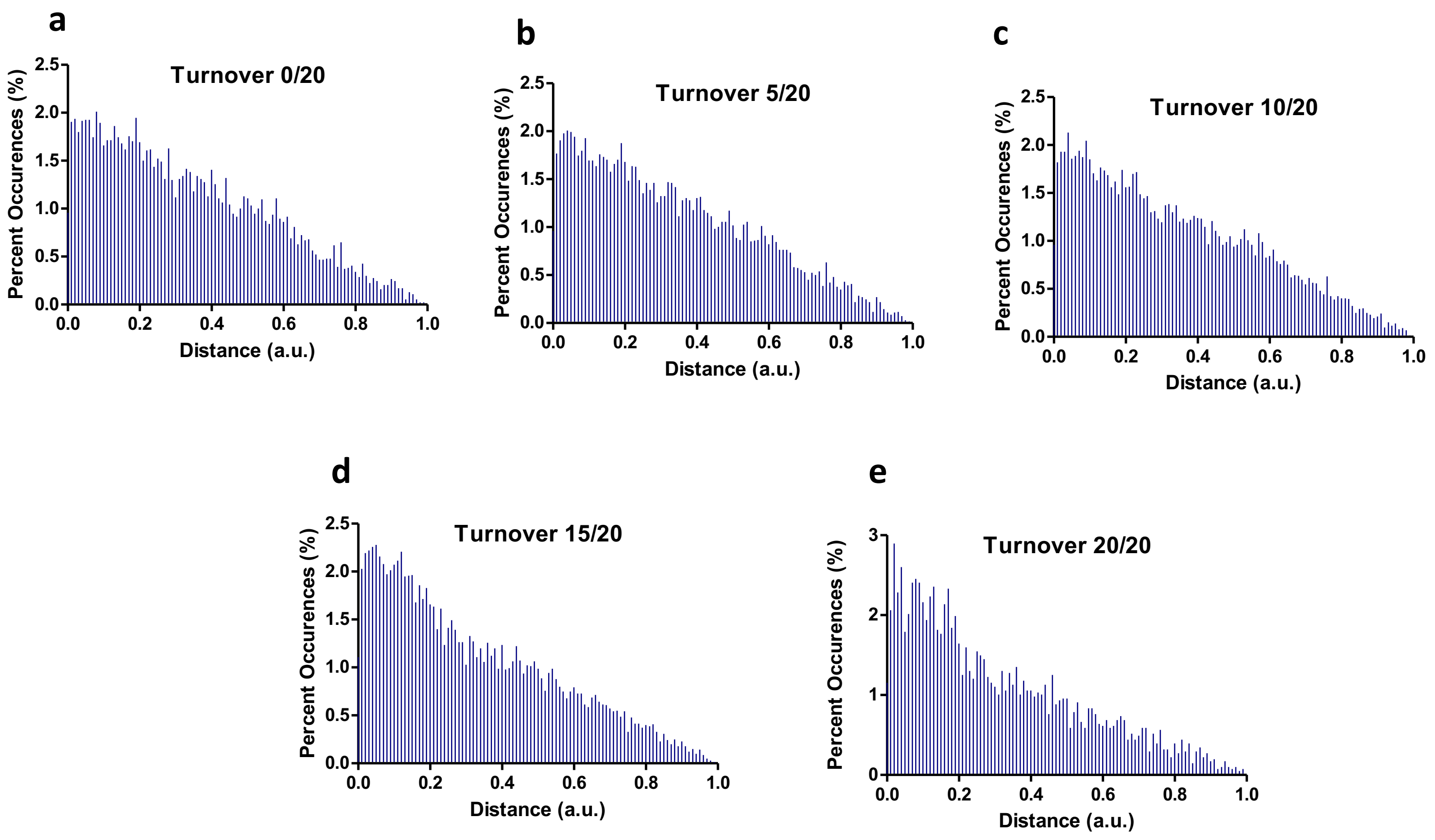


**Supplementary Figure 17. Distribution of distances between synapse pairs for different levels of synaptic turnover.** The number of dendritic subunits per neuron with high turnover rate was increased from 0 in (**a**) to 20 in (**e**). Only potentiated synapse pairs (synaptic weight >0.8) are shown. Distances are shown in arbitrary units (1 arbitrary unit is presumed to represent approximately ~50 μm dendritic length)

## Properties of Spine Clustering

| | Control | Trained | Trained |
|---|---|---|---|
| Learning Task | No training | Contextual Fear Conditioning | Morris Water Maze |
| Number of mice | 17 | 17 | 11 |
| Total Length of Dendrite (μm) | 13711.1 | 13002.8 | 6548.3 |
| Number of Dendritic Segments | 532 | 579 | 136 |
| Dendritic Segments per mouse (mean ± s.e.m.) | 31.29 ± 5.26 | 34.06 ± 3.69 | 12.36 ± 0.97 |
| Total Number of Spines at First Imaging Day | 6672 | 6297 | 2277 |
| Total Number of Spines at Last Imaging Day | 6649 | 6293 | 2273 |
| Spine Density at First Imaging Day (spines/μm) (mean ± s.e.m.) | 0.391 ± 0.01 | 0.397 ± 0.01 | 0.352 ± 0.01 |
| Spine Density at Last Imaging Day (spines/μm) (mean ± s.e.m.) | 0.390 ± 0.01 | 0.397 ± 0.01 | 0.348 ± 0.01 |
| Number of New, Stable Spines After Learning | 377 | 371 | 365 |
| Number of Clustered Spines | 110 | 167 | 163 |
| Directly Calculated Percentage of Clustered Spines | 29% | 45% | 45% |
| Average Percentage of Clustered Spines Across mice | 23% | 42% | 43% |

**Supplementary Table 1. Properties of spine clustering**

Supplementary Table 2

## Configurations of Spine Clustering

| Number of Spines in Cluster | Cluster Configurations | Number of Cases | | |
|---|---|---|---|---|
| | | Control | CFC | MWM |
| 2 | N-N | 26 | 28 | 33 |
| | N-S-N | 10 | 23 | 13 |
| | N-S-S-N | 3 | 6 | 4 |
| | N-S-S-S-N | 2 | 1 | 0 |
| | Total | 41 | 58 | 50 |
| | Percentage of all clusters | 82% | 78% | 72% |
| | Percentage of clustered spines | 74.5% | 69.4% | 61% |
| 3 | N-N-N | 2 | 8 | 4 |
| | N-N-S-N | 3 | 1 | 5 |
| | N-N-S-S-N | 1 | 1 | 1 |
| | N-S-N-S-N | 2 | 1 | 1 |
| | N-S-N-S-S-N | 0 | 1 | 0 |
| | N-S-S-N-S-S-N | 0 | 0 | 1 |
| | N-S-N-S-S-S-N | 0 | 1 | 1 |
| | Total | 8 | 13 | 13 |
| | Percentage of all clusters | 16% | 18% | 19% |
| | Percentage of clustered spines | 21.8% | 23.4% | 24% |
| 4 | N-N-N-N | 1 | 0 | 2 |
| | N-N-S-S-N-N | 0 | 1 | 0 |
| | N-S-N-N-S-N | 0 | 0 | 1 |
| | N-S-N-N-S-S-N | 0 | 1 | 1 |
| | N-S-N-S-S-S-N-N | 0 | 1 | 0 |
| | N-S-S-N-N-S-S-S-N | 0 | 0 | 1 |
| | N-S-S-N-S-S-N-S-N | 0 | 0 | 1 |
| | Total | 1 | 3 | 6 |
| | Percentage of all clusters | 2% | 4% | 9% |
| | Percentage of clustered spines | 3.6% | 7.2% | 15% |
| N=New, stable, clustered spine; S=Existing stable spine | | | | |
| CFC=Contextual fear conditioning; MWM=Morris water maze | | | | |

**Supplementary Table 2. Configurations of spine clustering**

## Stability of Spine Clusters and Spines by Cluster Size

| | Stable Clusters | Lost Clusters | Stable Spines | Lost Spines |
|---|---|---|---|---|
| 2-Spine Cluster | 33 | 6 | 46 | 32 |
| 3-Spine Cluster | 10 | 0 | 21 | 9 |
| 4-Spine Cluster | 1 | 0 | 3 | 1 |

**Supplementary Table 3. Stability of spine clusters and spines by cluster size**. Stable clusters are those that have at least one spine remaining at re-imaging at 4-6 weeks, while lost clusters are those that have no spines remaining at re-imaging at 4-6 weeks. Stable spines and lost spines represent the total number of spines present or lost, respectively, for each cluster size (i.e. 46 spines were stable across all clusters initially involving 2 spines). There is no significant difference in spine stability between cluster sizes (Fisher's exact test, $p$=0.5237), and no significant difference in cluster loss between cluster sizes (Fisher's exact test, $p$=0.4048).

Supplementary Table 4

# Parameters of Computational Model

| Parameter | Description | Value |
|---|---|---|
| $\tau_b$ | Passive dendritic integration time constant | 20 msec |
| $E_{syn}$ | Maximum unitary EPSP | 4.0 mV |
| $\theta_{dspike}$ | Depolarization threshold for dendritic spiking | 30 mV |
| $V_{dspike}$ | Dendritic spike maximum depolarization | 50.0 mV |
| $E_L$ | Somatic leakage reversal potential | 0 mV |
| $\Theta_{soma}$ | Voltage threshold for somatic spikes | 20 mV |
| $G_{syn}$ | Dendritic coupling constant | 20 pS |
| $C$ | Membrane capacitance | 200 pF |
| $g_L$ | Leak conductance | 6.67 nS |
| $\tau_{AHP}$ | Adaptation time constant of excitatory neurons | 180 msec |
| $\tau_{AHP,I}$ | Adaptation time constant of interneurons | 70 msec |
| $a_{AHP}$ | Adaptation conductance increase after a spike | 0.18 nS |
| $E_K$ | Adaptation reversal potential | -10 mV |
| $\tau_{bAP}$ | Back propagating action potential time constant | 15 msec |
| $E_{bAP}$ | Back propagating action potential max amplitude | 30 mV |
| $a_{Ca}$ | Calcium influx rate | 0.1 $msec^{-1}$ |
| *synTag (x)* | Value of synaptic tag as a function of Calcium Level *x* (Calcium control model) | $\left(\frac{1.3}{1+exp(-10(10x-3.5.))}\right)-\left(\frac{0.3}{1+exp(-19(10x-2.0))}\right)$ |
| $\Theta_{PRP}$ | Calcium threshold for PRP synthesis near a synapse | 2.0 (arbitrary units) |
| $\tau_{PRP}$ | Time constant for PRP level decay | 60 minutes |
| $\tau_H$ | Time constant of homeostatic synaptic scaling | 7 days |
| $W_{init}$ | Initial synapse weight | 0.2 |
| $\Theta_{removal}$ | Probability of turnover of a weak synapse (weight < $w_{init}$) | 0.79 per day |
| $N_{pyr}$ | Number of excitatory neurons | 240 |
| $N_{inh}$ | Number of inhibitory neurons | 60 |
| $N_{branches}$ | Number of branches per excitatory neuron | 20 |
| $N_{pyr \rightarrow inh}$ | Total number of random connections from pyramidal neurons to inhibitory neurons | 3480 |
| $N_{inh \rightarrow pyr}$ | Total number of random connections from inhibitory neurons to pyramidal neurons | 5760 |
| $N_{stim}$ | Total number of presynaptic memory-encoding input neurons | 100 |
| $N_{stim,memory}$ | Number of presynaptic input neurons that are active for one encoded memory | 10 |
| $N_{stim \rightarrow pyr}$ | Number of synapses from memory-encoding neurons to random excitatory dendritic subunits | 48000 |

**Supplementary Table 4. Parameters of computational model**
